# Epitope-Containing Short Peptides Capture Distinct IgG Serodynamics That Enable Differentiating Infected from Vaccinated Animals for Live-Attenuated Vaccines

**DOI:** 10.1128/JVI.01573-19

**Published:** 2020-02-28

**Authors:** Qinghong Xue, Hongke Xu, Huaidong Liu, Jiaojiao Pan, Jiao Yang, Miao Sun, Yanfei Chen, Wenwen Xu, Xuepeng Cai, Hongwei Ma

**Affiliations:** a China Institute of Veterinary Drug Control, China; b Division of Nanobiomedicine, Suzhou Institute of Nano-Tech and Nano-Bionics, Chinese Academy of Sciences, China; St. Jude Children's Research Hospital

**Keywords:** epitope, peptide microarray, vaccine

## Abstract

Differentiating infected from vaccinated animals (DIVA) strategies have been central enabling techniques in several successful viral disease elimination programs. However, owing to their long and uncertain development process, no DIVA-compatible vaccines are available for many important diseases. We report herein a new DIVA strategy based on hybrid protein-peptide microarrays which can theoretically work with any vaccine. Leading from our findings from peste des petits ruminants (PPR) virus, we found 4 epitope-containing short peptides (ECSPs) which have distinct IgG serodynamics: anti-ECSP IgGs only exist for 10 to 60 days postvaccination (dpv), while anti-protein IgGs remained at high levels for >1,000 dpv. These data enabled the design of a DIVA diagnostic microarray containing 4 ECSPs and 3 proteins, which, unlike competitive enzyme-linked immunosorbent assay (cELISA) and virus neutralization tests (VNTs), enables ongoing monitoring of serological differences between vaccinated individuals and individuals exposed to the pathogen. For 25 goats after 60 dpv, 13 were detected with positive anti-ECSP IgGs, indicating recent infections in vaccinated goat herds. These DIVA diagnostic microarrays will almost certainly facilitate eradication programs for (re)emerging pathogens and zoonoses.

**IMPORTANCE** Outbreaks of infectious diseases caused by viruses, such as pseudorabies (PR), foot-and-mouth disease (FMD), and PPR viruses, led to economic losses reaching billions of dollars. Both PR and FMD were eliminated in several countries via large-scale vaccination programs using DIVA-compatible vaccines, which lack the gE protein and nonstructural proteins, respectively. However, there are still extensive challenges facing the development and deployment of DIVA-compatible vaccines because they are time-consuming and full of uncertainty. Further, the negative marker strategy used for DIVA-compatible vaccines is no longer functional for live-attenuated vaccines. To avoid these disadvantageous scenarios, a new strategy is desired. Here, we made the exciting discovery that different IgG serodynamics can be monitored when using protein-based assays versus arrays comprising ECSPs. This DIVA microarray strategy should, in theory, work for any vaccine.

## Abstract (precis)

Outbreaks of infectious diseases caused by viruses, such as pseudorabies (PR), foot-and-mouth disease (FMD), and PPR viruses, led to economic losses reaching billions of dollars. Both PR and FMD were eliminated in several countries via large-scale vaccination programs using DIVA-compatible vaccines, which lack the gE protein and nonstructural proteins, respectively. However, there are still extensive challenges facing the development and deployment of DIVA-compatible vaccines because they are time-consuming and full of uncertainty. Further, the negative marker strategy used for DIVA-compatible vaccines is no longer functional for live-attenuated vaccines. To avoid these disadvantageous scenarios, a new strategy is desired. Here, we made the exciting discovery that different IgG serodynamics can be monitored when using protein-based assays versus arrays comprising ECSPs. This DIVA microarray strategy should, in theory, work for any vaccine.

## INTRODUCTION

The management of viral diseases in both humans and veterinary medicine involves a series of successive steps, including identification of the causal virus by researchers, treatment by clinicians and veterinarians, public health and agricultural management of active infections (including quarantine and culling), the development and deployment of vaccines to prevent further infections in the population, technologies to enable ongoing surveillance of new viral infections and immunization efficacy, and ultimately, the elimination or even global eradication of a particular pathogen. The only two pathogens for which all of these steps have been accomplished are the smallpox and rinderpest viruses (1, 2).

However, a notable unintended consequence is that rinderpest eradication may have facilitated the spread of another major viral pathogen, the peste des petits ruminants virus (PPRV), among sheep and goat herds across Africa and Asia (3). Animals exposed to a vaccine against rinderpest are known to become immune to PPRV (4), so the cessation of vaccination upon virus eradication suddenly exposed millions of animals to this less deadly but still extremely economically damaging viral disease. Indeed, the most recent global pandemic of PPRV coincided with the vaccination cessation phase of the rinderpest eradication program, a situation that has—based on extensive work by scientists in many national-level programs—led to PPRV’s status as the recognized likeliest candidate for the third successful virus eradication (5). Unlike the conspicuously symptomatic smallpox disease and the extremely high lethality of rinderpest, a major challenge for PPR eradication efforts has been the need for ongoing surveillance in herds. In particular, the identification of active infections and monitoring of postvaccination immune status within populations that have already been vaccinated is recognized as the primary remaining barrier to PPR eradication.

Upon deciding to attempt the elimination of a virus from a particular geographical area, policy managers must select a means to accomplish ongoing surveillance of viral infections in a vaccinated population. Ideally, such a surveillance method would enable monitoring the presence of the virus in a way that distinguishes the serological differences between vaccinated individuals and individuals that have actually been exposed to the pathogen; this would allow for the identification of the precise location where a reemergence of a pathogen is occurring. To this end, there is a suite of technologies and strategies that are broadly known as “differentiating infected from vaccinated animals” (DIVA) (6). A pillar of DIVA strategies employed to date, for example, in pseudorabies (PR) (7) and foot-and-mouth disease (FMD) elimination programs (8), has been the need to develop both an effective DIVA-compatible vaccine (also known as a “marker” or “tagged” vaccine) and a serological test that can differentiate between vaccine-induced antibodies and antibodies against the current field strain of the virus. However, DIVA-compatible vaccines are often not as effective as conventional vaccines, and their development is both time-consuming and full of uncertainty (9).

For the aforementioned PPRV, the Office International Des Epizooties currently recommends two methods for PPRV diagnosis: a competitive enzyme-linked immunosorbent assay (cELISA) and a virus neutralization test (VNT) (10); unfortunately, both of these are incompatible with a DIVA strategy. Further, there are extensive challenges facing the development and deployment of DIVA-compatible vaccines and corresponding monitoring: (i) taking PR as an example, when a new mutated virus emerges that can escape from the protection provided by the DIVA-compatible vaccine (11), the development of a new DIVA-compatible vaccine would not be a timely and economically wise choice; (ii) taking FMD as an example, there will be detectable anti-nonstructural protein (NSP) IgGs after multiple rounds of vaccination due to residual NSPs in the vaccine, causing a false infection result (12); and (iii) taking PPR as an example, the negative marker strategy used for DIVA-compatible vaccines is no longer functional for live-attenuated vaccines. To avoid these disadvantageous scenarios, a new strategy is desired.

Peptide microarrays were first developed in the early 1980s (13) after the invention of solid-phase synthesis of peptides, a breakthrough that won the Nobel Prize for Chemistry in 1984. After years of exploration, some technical difficulties and high costs have limited the popular application of peptide microarrays to applications such as pharmacological epitope discovery. While working with very-low-background-signal peptide microarray technology (14, 15), we recently discovered that epitope-containing short peptides (ECSPs) capture different IgG serodynamics than do protein-based assays. In this report, through longitudinal sera analysis with a PPRV-originated peptide microarray, commercial cELISA, and VNT, we identified 4 dominant ECSPs and confirmed that the distinct IgG serodynamics we initially observed also occur in goats that have been vaccinated with a PPR live-attenuated vaccine. Building from this, we illustrate an entirely new DIVA strategy based on these hybrid ECSP and protein microarrays that, importantly, no longer requires the development of a DIVA-compatible vaccine or the need for negative marker-specific serological monitoring technology. Our work thus opens a large new opportunity for epizoology and zoonoses, raising the possibility that all vaccines can potentially be used in DIVA strategies to facilitate elimination and eradication programs.

(This article was submitted to an online preprint archive [16].)

## RESULTS AND DISCUSSION

**FIG 1:**
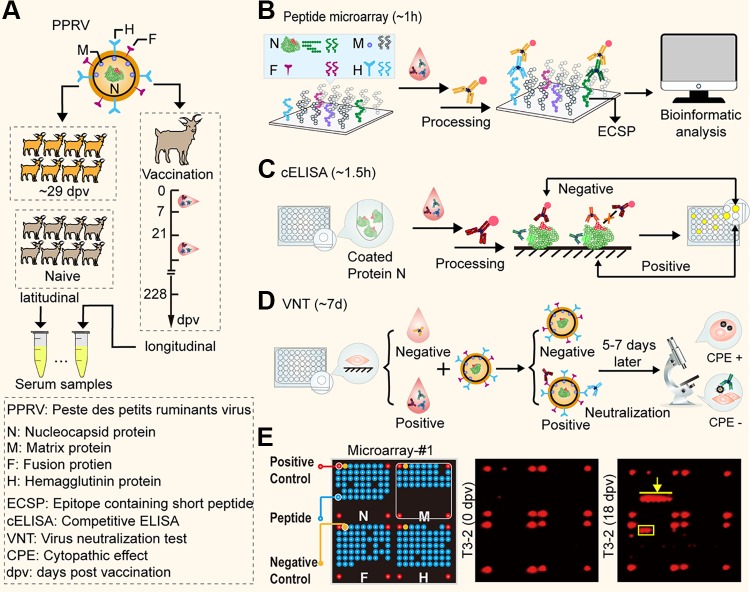
Screening for ECSPs that possess both unique IgG serodynamics and diagnostic potential. (A) Longitudinal samples were prepared through immunization of goats with a live-attenuated vaccine, and blood samples were collected at specific intervals. Latitudinal samples included vaccinated sera and negative sera from naive goats without vaccination or infection. (B) Overview of screening with the peptide microarray platform; it is a modified indirect ELISA configured with chemiluminescence, and the whole test takes ∼1 h. (C) Workflow of cELISA (∼1.5 h) and (D) VNT (∼7 days). (E, Left) Schematic of microarray 1. It constitutes four subarrays; each subarray contains four positive control points (red, goat IgG), one negative control point (yellow, printing buffer), and 47, 31, 49, and 56 20-mer peptides (blue) from the N, M, F, and H proteins of PPRV, respectively. The missing blue dots within each subarray indicate that the corresponding peptides were not able to be synthesized. (E, Middle and Right) Two representative chemiluminescence images from the peptide microarray with longitudinal samples at 0 dpv (middle) and 18 dpv (right) from the T3-2 goat.

Aiming to recapture the different IgG serodynamics detected by protein-/virus- or short peptide-based assays, we first prepared longitudinal sera by immunizing 9 goats with a live-attenuated vaccine in the laboratory (Fig. 1A). The sera were then evaluated using three different analytical platforms, namely, our peptide microarray (microarray 1), commercial cELISA, and VNTs (Fig. 1B to D). Compared to the single (aggregate) index value from a protein0based assay (17), microarray 1, with its 183 peptides, would produce 183 separate indices from one sample at a given time point (Fig. 1E, left), which is obviously more informative than the other platforms regarding IgG composition in sera. cELISA uses a purified recombinant N protein as the antigen (18) and a specific monoclonal antibody, binding to the domain on the amino-terminal half (19), as the detection antibody (Fig. 1C). The VNT detected antibodies against the F or H proteins that were exposed on the surface of PPRV (Fig. 1D) (20).

**FIG 2:**
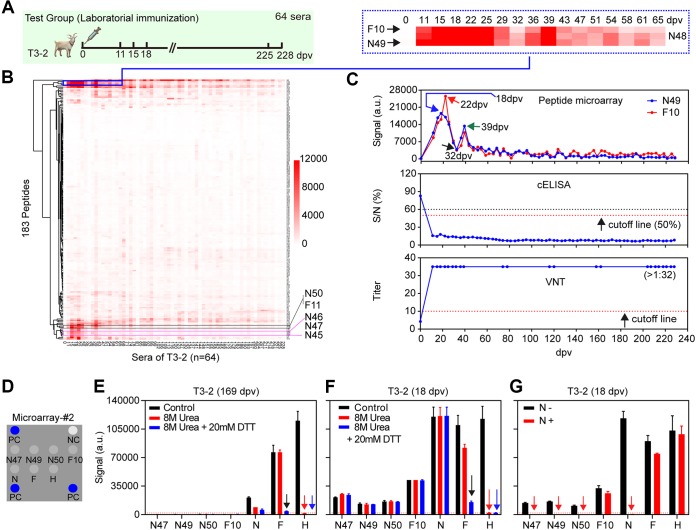
Distinct serodynamics and epitope type changes of IgGs. (A) Sixty-four longitudinal sera collected from a representative goat (T3-2). (B) The clustered heat map of the 64 longitudinal sera from T3-2 against 183 peptides (microarray 1). (C) Comparison of the IgG serodynamics from 64 longitudinal sera of T3-2 detected by ECSPs (N49 and F10), the cELISA kit, and VNT, respectively. (D) The layout of microarray 2. PC is the goat IgG, and NC is the printing buffer; these are the positive and negative controls, respectively, for horseradish peroxidase (HRP)-conjugated antibody interaction. (E) Results of serum T3-2 (169 dpv) and (F) T3-2 (18 dpv) incubated with microarray 2 after different denaturing treatments. (G) Blocking experiment with N protein incubated with T3-2 (18 dpv). Treatment with 20 mM DTT alone showed no signal change on any of the ECSPs and proteins (data not shown).

Subject T3-2 was chosen as a representative case based on its relatively higher signal level indicated by the results of microarray 1 (Fig. 2A). Compared to the negative control serum sampled at 0 days postvaccination (dpv) (Fig. 1E, middle), two groups of peptides, N45 to N50 from the N protein (Fig. 1E, right, yellow arrow) and F9 to F11 from the F protein (Fig. 1E, right, yellow box), were observed over time during the immune response. A large-scale analysis with a clustered heat map (Fig. 2B) clearly demonstrates that these peptides contain linear B-cell epitopes. No such peptides were found for the M or H proteins. All 8 other goats showed the same trend, with only minor dissenting features due to individual immune differences (21) (data not shown), so we henceforth focused on N45 to N50 and F9 to F11 as ECSPs.

Previous studies have shown that the PPR live-attenuated vaccine can induce lifelong immunity (22). Our results showed that the titers of neutralization antibodies detected by VNT and the nonneutralization IgGs detected by cELISA were maintained at constantly high levels from 11 dpv to 228 dpv (Fig. 2C, middle and bottom). However, the IgG serodynamics detected by ECSPs, such as N49 or F10, were dramatically different from cELISA and VNT; with ECSP, we noted a bimodal curve with two peaks at the early stage (Fig. 2C, top). Specifically, the signal for the anti-N49 IgGs was undetectable at 0 dpv and reached an initial peak at 18 dpv (blue arrow; signal, 18,524 [expressed in a.u.]) and then declined to a low but detectable level at 32 dpv (black arrow; signal, 3,800) before reaching a second but lower peak at 39 dpv (green arrow; signal, 13,372) and finally declining dramatically to below the detection limit by around 60 dpv. The activity of anti-F10 IgGs was similar to anti-N49 IgGs, but reached its first peak at 22 dpv, 4 days later than the anti-N49 IgGs.

**FIG 3:**
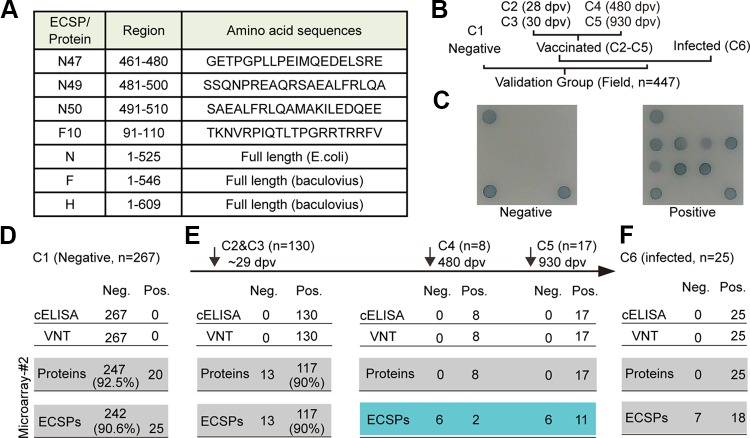
Validation of distinct IgG serodynamics, using latitudinal sera, which have implications for vaccine titer evaluation and DIVA. For individual ECSPs or proteins of microarray 2, the result was positive if its S/P was larger than the cutoff. For the ECSP combination (i.e., N47 + N49 + N50 + F10), the result was positive if any ECSP was positive. For microarray 2, the result was positive if any two proteins were positive (mainly for titer evaluation) or if any ECSP was positive (mainly for DIVA). (A) Selected ECSPs and proteins. (B) Six groups of sera (C1 through C6) were collected to test the microarray 2. The positive/negative status of these latitudinal samples were confirmed by both cELISA and VNT. (C) Representative images of negative and positive serum. Results of sera against microarray 2 are as follows: (D) C1, the 267 negative sera. (E, Left) One hundred thirty positive sera (C2 and C3 combined). (E, Middle) C4, 8 positive sera at 480 dpv. (E, Right) C5, 17 positive sera at 930 dpv. (F) C6, 25 positive sera due to virus infection.

**FIG 4:**
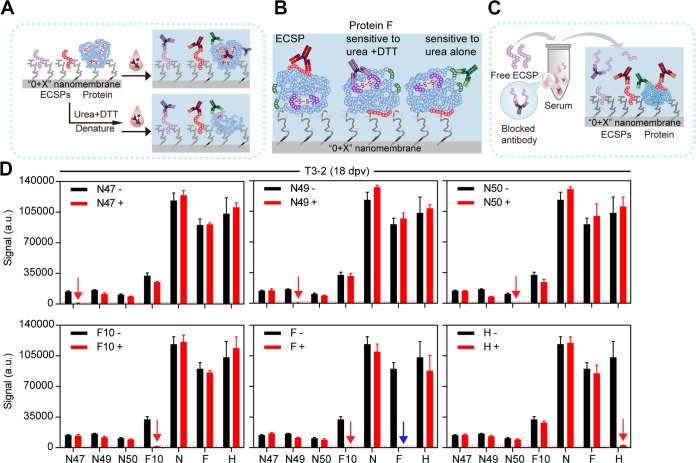
Denaturation and blocking experiments. (A) Flowchart of denaturation experiments (see Fig. 2E and F for data). (B) Three groups of anti-F IgGs. (C) Flowchart of blocking experiments. Free ECSP or protein was individually doped to serum before screening against microarray 2. (D) Results of blocking experiments.

Given that the results from microarray 1, cELISA (protein N), and VNT (mainly proteins F and H at the surface of PPRV) cannot be compared quantitatively, we printed 4 ECSPs and 3 proteins collectively to form microarray 2 (Fig. 2D and Fig. 3A) for further tests. To explore the position of the ECSPs on the proteins, we performed blocking experiments (Fig. 4C), and the results not only confirmed their specificity but also supported that the three N-protein ECSPs and F10 are located on the surface of proteins N and F. For example, free ECSP N47 blocked the signal of N47 in microarray 2 but did not block other ECSPs or proteins (Fig. 4D). Additionally, since free protein N was able to block both protein N and the ECSPs which originated from protein N in microarray 2, we concluded that the C terminus of protein N is rich in linear epitopes, similar to protein N of the measles virus (MeV); both viruses belong to the same *Morbillivirus* genus (23) (Fig. 2G and Fig. 4D).

The different IgG serodynamics detected by ECSPs and cELISA/VNT may be attributed to the short and long durations of IgGs that recognize linear and conformational epitopes, respectively. To probe the nature of interactions between ECSPs/proteins and IgGs at different immunization stages, prior to incubation with serum, we used the disulfide-reducing agent dithiothreitol (DTT) and protein denaturant urea to disrupt conformations of printed proteins in microarray 2 (Fig. 4A).

First, using intact proteins as control, without denaturing treatment (Fig. 2E and F, black columns) gave a quantitative comparison of various IgG levels. A later-stage serum T3-2 (169 dpv) lacked anti-ECSP IgGs but contained different levels of anti-protein IgGs (Fig. 2E). Signals due to anti-H, anti-F, and anti-N IgGs were ∼115,300, ∼76,600, and ∼20,950, respectively. An early-stage serum T3-2 (18 dpv) contained IgGs for both ECSPs and proteins (Fig. 2F). Signals for anti-ECSPs ranged in intensity from 13,000 to 42,500; we also measured the signals for anti-H (∼118,000), anti-F (∼109,900), and anti-N IgGs (∼120,200).

These quantitative data implied that proteins N, F, and H can outcompete ECSPs. At the early stage, proteins induced 3 to 10 times more IgG production than ECSPs (assuming all IgGs have the same affinity such that the detected signal depends solely on concentration). At the later stage, proteins induced long-lived plasma cells (LLPCs) for a continuous supply of IgGs, which is the key to obtaining lifelong immunity. While the anti-N IgG level dropped dramatically (from ∼120,200 to ∼20,950), the anti-H IgG level was almost constant (∼115,300 versus ∼118,000), and the anti-F IgG level dropped slightly from ∼109,900 to ∼76,600, implying the immune system was able to selectively focus on the production of functional (i.e., neutralization) IgGs (17).

Second, printed proteins denatured by combined urea and DTT (hereafter, urea+DTT) treatment were used to infer whether the epitope type of IgG changed during the course of the immune response (Fig. 2E and F, blue columns). For a later-stage serum T3-2 (169 dpv), the urea+DTT treatment caused a significant signal loss for proteins N (from ∼20,950 to ∼5,900), H (∼115,300 to 125), and F (∼76,600 to ∼4,300) (Fig. 2E). Such sensitivity to protein conformational changes can be explained by assuming there were only IgGs that recognize conformational epitopes on proteins N, H, and F (or perhaps other IgGs were present but below the detection level). In contrast, for an early-stage serum T3-2 (18 dpv) (Fig. 2F), we found that none of the four ECSPs exhibited any obvious signal change after urea+DTT treatments, which agreed with the fact that short peptides (20-mer in this paper) have no conformation *per se* and are thus apparently insensitive to urea+DTT treatments. Three proteins, however, behaved differently: proteins N and F had both IgGs for linear and conformational epitopes; protein H had IgGs only for conformational epitopes.

For protein N, almost no signal loss was observed after urea+DTT treatments (Fig. 2F). This finding suggested that either the conformational structure of the N protein was not disrupted completely or there were abundant IgGs, which can recognize the denatured N protein (i.e., linear epitopes) to compensate for the loss of signal from IgGs, which are sensitive to conformation. The latter scenario is more likely, given that the primary structure of protein N contains only one cysteine in its full length and considering that this serum sample represents the peak of the IgG serodynamics of ECSPs, thus likely possessing abundant IgGs, which can recognize both linear and conformational epitopes.

For protein F, the signal dropped from 109,918 to 15,312 with the urea+DTT treatments (Fig. 2F). This significant remaining signal indicated the existence of IgGs that recognize linear epitopes, such as F10. For protein H, a >98% loss of signal from the H protein after urea+DTT treatments suggested that the H protein probably has no linear epitopes (Fig. 2F).

Third, printed proteins denatured by urea treatment alone indicated the IgG epitope-type changes for the three proteins were different (Fig. 2E and F, red columns). Proteins N and H showed no differences in signal loss for either urea or urea+DTT treatments (Fig. 2E and F, red and blue columns). Protein F, however, responded differently to urea and urea+DTT treatments: the signal dropped from ∼109,900 to ∼82,100 after urea treatment and dropped to ∼15,300 with the urea+DTT treatments (Fig. 2F). We therefore propose that at the early stage, there were three groups of IgGs (Fig. 4B): one is sensitive to conformation and can be disrupted by urea alone (signal contribution, 25%), one is sensitive to conformation and can be disrupted by the urea+DTT treatment (signal contribution, 86%), and the final one is for linear epitopes (signal contribution, 14%). For the later-stage serum (Fig. 2E), the urea+DTT treatment led to almost complete signal loss, indicating that there were IgGs for conformational but not linear epitopes, while the treatment of urea alone showed no signal loss, indicating that there were no IgGs for conformational epitopes sensitive to urea alone.

This conformational sensitivity of IgGs to the F and H proteins implies that these IgGs are very likely responsible for the neutralization function that we observed in the VNT results. These results are in agreement with the current model of IgG development as elicited by immunization (17, 24, 25): at the early stage, immunization-elicited IgGs can recognize both linear (ECSPs and proteins) and conformational epitopes (proteins). As the immune system continues to interact with the antigen (vaccine), the host is able to, via an unknown mechanism, concentrate its resources to produce a few dominant IgGs that are functional-neutralization IgGs for conformational epitopes present at the surface of a virus. We should therefore be able to both detect IgG epitope-type changes and monitor different IgG serodynamics occurring between ECSPs and proteins over time during an immune response.

Due to the limited number of experimental goats, the longitudinal sera group only had a small number of samples at each time point (3 samples for the T3 group). Attempting to extend our findings regarding IgG serodynamics, we tested more latitudinal field sera at several time points of their serodynamic curves, namely, the C1 to C6 groups (Fig. 3B), which also served as the verification/validation groups for microarray 2. Furthermore, 3,3′,5,5′-tetramethylbenzidine (TMB) was used to replace the chemiluminescence substrate, so the microarray was more affordable for field use. A testing procedure was established with positive and negative control sera included in the test, and the signals of individual ECSPs or proteins were further converted to the ratio of the sample to the positive control (S/P). The cutoff S/P for each ECSP or protein was determined through receiver operating characteristic (ROC) curve analysis based on 267 negative sera (C1) and 130 vaccinated sera at ∼29 dpv.

According to the aforementioned IgG serodynamics, C1 to C3 (early-stage sera, <60 dpv) should have the same result pattern from both ECSPs and proteins. Two hundred sixty-seven negative sera (C1) were tested for their relative specificity. The relative specificity for the ECSP combination was 90.6% but reached 92.5% for the protein combination (Fig. 3D), both meeting the industrial standard of specificity, i.e., >90%. For the 20 samples with discrepancy (false positive), 10 of them were in the gray zone (near the cutoff line).

For the C2 and C3 groups (∼29 dpv), although they were ∼1 week behind the peak of the IgG serodynamic curves of anti-ECSPs, we still obtained a relatively high sensitivity of 90% for ECSPs (we believe the sensitivity may be higher than 90% with samples ∼20 dpv). The relative sensitivity using the protein combination was also 90% (Fig. 3E, left). Through the C1 to C3 groups, we confirmed that the behavior of both ECSPs and proteins in microarray 2 agreed with the IgG serodynamic trends. Thus, microarray 2 is suitable for titer evaluation of the PPR live-attenuated vaccine.

According to the aforementioned IgG serodynamics, groups C4 to C5 (later-stage sera, >60 dpv) would be expected to have different results between ECSPs and proteins. Thus, we present the results of microarray 2 for these groups in the following format: proteins/ECSPs. For C4 (480 dpv) and C5 (930 dpv), one anticipated +/− based on the IgG serodynamic curves, but 2/8 and 11/17 sera for C4 and C5, respectively, showed +/+ (Fig. 3E, right). Of the total 25 serum samples, these 13 were confirmed by microarray 1, implying these goats were infected by PPRV within the last 60 days before sampling. The agreement of both microarrays (1 and 2) suggested that ECSPs captured newly elicited IgGs, while the proteins capture IgGs secreted by both short-lived plasma cells (SLPCs) and LLPCs. Thus, we hereafter refer to microarray 2 as the DIVA microarray. For the 25 sera from goats infected by PPRV (as confirmed by both cELISA and VNT) (C6), we did not know the exact date of infection. Eighteen out of twenty-five were +,+, indicating recent infection (<60 days postinfection [dpi]); 7/25 were +/−, indicating an infection time point >60 dpi (Fig. 3F). Note that this timing information is provided by the DIVA microarray but not cELISA or VNT.

**FIG 5:**
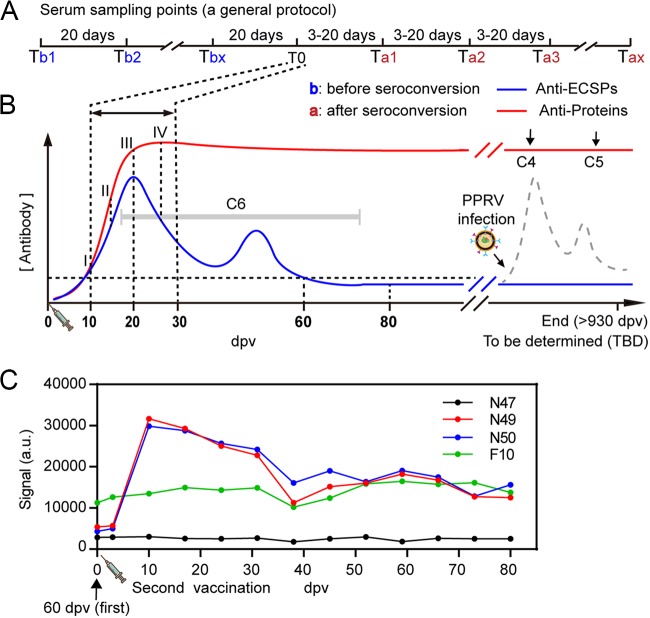
A general protocol for multiple purposes: vaccine titer evaluation, DIVA diagnosis, and surveillance as suitable application scenarios. (A) Proposed serum sampling schedule. (B) Serum sampling points matched with IgG serodynamics. (C) With use of the PPR live-attenuated vaccine to mimic the virus infection, the spiked ECSPs response clearly indicated an infection.

To further examine the diagnostic and DIVA potential of microarray 2 with larger sample sizes and inspired by our results for the C4 to C6 groups, we designed a general program (Fig. 5A) that realizes DIVA surveillance for both a well-managed herd and a poorly managed herd/wildlife. For the well-managed herd, one knows the vaccination date, so blood sampling started from Tbx (x = 1, b stands for before T0). The results obtained as proteins/ECSPs for sera against the DIVA microarray should follow the curves in Fig. 5B. For the period of 0 to 60 dpv, the result could also be used to evaluate the vaccination titer. The interval between two Ta dates (a stands for after T0) could be adjusted as needed from 3 to 20 days. After 80 dpv, for example, results of +/+ or +/− would indicate, respectively, the presence or absence of infection (Fig. 5B).

For a poorly managed herd, if the vaccination date was unknown (or not vaccinated), represented hypothetically as the gray band of C6 in Fig. 5B, a randomly picked date could be used as Tb1. The T0 could be anywhere from 10 to 28 dpv, partially affected by the individual immune status (21). We recommended a 3- to 5-day interval between T0 and Ta1 and Tax (x = 60/interval), so a detailed IgG serodynamic curve could be obtained, which would be informative for deducing the position T0.

As a mimic of virus infection, a second immunization (with 50 doses) was carried out on goats at 60 dpv from the first immunization (Fig. 5C). Although given as a 50-fold increase in dosage, the anti-protein IgG level remained the same (signal, ∼120,000). ECSPs N49 and N50 peaked at 10 dpv of the second immunization and waned monotonically. The signal did not reach zero, even at 80 dpv, which was attributed to the 50-fold increase in dosage.

Since the live-attenuated vaccine is closest to the situation of wild virus infection, this DIVA microarray strategy should, in theory, work for any vaccine: the protein section detects long-lasting IgGs (same function as cELISA and VNT), and the ECSP section determines the stage of vaccination/infection (the new function that is vital for DIVA). When these two sections are combined, this microarray realizes DIVA. Our ongoing efforts are to translate this DIVA microarray strategy to fight emerging infections and zoonoses (26–28). As more vaccines are developed to prevent human diseases, e.g., human papillomavirus vaccines for cervical cancer (29) and enterovirus 71 vaccines for hand, foot, and mouth disease (HFMD) (30), there is an increasing demand for people to differentiate infection from vaccination. We noticed that the IgG serodynamics of most human vaccines have been developed based on data from protein- or virus-based assays (31–33). If short peptides share a similar trend in human vaccines, i.e., they wane monotonically, then obviously, a similar strategy could be constructed based on our peptide microarray technology, which would be useful in preventing the transmission of the field strain of a virus. This work also implies that applications of these peptide microarrays should provide access to a new world of vaccines and diagnostics for viral diseases.

## MATERIALS AND METHODS

### Ethics statement.

The protocol of the animal study was approved by the Committee on the Ethics of Animal Experiments of the China Institute of Veterinary Drug Control (permit number [2016]00114). The study was conducted following the Guide for the Care and Use of Animals in Research of the People’s Republic of China.

### Vaccine and vaccination.

The PPR vaccine is produced by Tiankang Co., Ltd and is a live vaccine based on the Nigeria 75/1 attenuated strain (lineage II). The virus titer was 3.2 log_10_ TCID50/dose of 1 ml. PPR-capripox live vaccine is produced by Sinopharm Yangzhou VAC Biological Engineering Co., Ltd. The PPR virus titer was 3.3 log_10_ 50% tissue culture infective dose (TCID_50_)/dose of 1 ml, and the POX virus titer was 3.6 log_10_ TCID_50_/dose of 1 ml. To evaluate the IgG serodynamics elicited by the vaccine, 3 healthy and susceptible goats (T1) were immunized with the PPR vaccine through a 1-ml subcutaneous injection in the neck, 3 goats (T2) were also immunized with the PPR vaccine, and 3 goats (T3) were immunized with PPR-capripox vaccine through 1-ml subcutaneous injection in the neck.

### Serum samples.

Longitudinal sera were prepared from 9 goats in the lab. Three goats (T1) were sampled daily from 0 to 21 dpv, 3 (T2) were sampled from 0 to 44 dpv at 3- to 4-day intervals, and 3 (T3) were sampled from 0 to 228 dpv at 3- to 4-day intervals. All samples were prepared as follows: 500 to 1,000 μl of whole blood from the jugular vein was collected with a disposable container and statically placed at 4°C for 2 h; then the sample was centrifuged at 2,000 rpm for 15 min, and the supernatant was inactivated in a water bath at 56°C for 30 min and finally stored at −80°C.

Latitudinal sera were collected from the field and stored in the lab, including 267 negative samples (negative neutralization antibody) from healthy goats (C1), 25 positive samples from vaccinated goats at 28 dpv (C2), 105 positive samples from vaccinated goats at 30 dpv (C3), 8 positive samples from vaccinated goats at 480 dpv (C4), 25 positive samples from vaccinated goats at 930 dpv (C5), and 25 positive samples from infected goats (C6).

### Virus and infection.

To explore the antibody titer variation of the infected goat, a second vaccination with a live-attenuated vaccine (single vaccine) that mimicked the infection was carried out on the first vaccinated goat (PPR-capripox vaccine) at a 50-goat dose was administered to vaccinate three corresponding goats.

### Peptide library.

The proteins of N (GenBank accession no. CAA52454.1), M (GenBank accession no. CAJ01698.1), F (GenBank accession no. CAJ01699.1), and H (GenBank accession no. CAJ01700.1) from PPR vaccine strain Nigeria 75/1 (GenBank accession no. X74443.2) were employed to analyze the amino acid (aa) sequences. Twenty-mer peptides with an overlap of 10-aa residues covering the entire protein were chemically synthesized by GL Biochem (Shanghai, China), which finally yielded 183 peptides in total, including 47 peptides from N (N1 to N52; N27, N32, N34, N51, and N52 failed to be synthesized), 31 from M (M1 to M33; M6 and M14 failed to be synthesized), 49 from F (F1 to F54; F12, F30, F32, F39, and F50 failed to be synthesized), and 56 from H (H1 to H60; H4, H5, H33, and H46 failed to be synthesized), respectively.

### Microarrays.

Microarray 1 (peptide microarrays) with a whole panel of 183 peptides was fabricated as previously reported (34). Briefly, ∼0.6 nl of each peptide with a concentration of 0.1 mg/ml was printed onto the activated nanomembranes by contact spotter Smart 48 (Capital Bio, Beijing, China) to form 9 by 9 by 4 microarrays (Fig. 1E, left). In each subarray, there are four positive controls printed with goat IgG at a concentration of 50 μg/ml and one negative control with printing buffer.

Microarray 2, which includes four ECSPs (N47, N49, N50, and F10), three proteins (N, F, and H), and goat IgG (i.e., three positive control points) was printed in a 4 by 4 array by the noncontact spotter sciFlexarrayer S1 (Scienion, Berlin, Germany) (Fig. 2D), with a concentration of 0.1 mg/ml for each peptide or protein and 50 μg/ml for goat IgG. N was purchased from Suzhou Epitope Biotech Ltd. Co., China. Proteins F and H were purchased from Novo Biotech, China.

### Serum screening with microarray 1 (test group).

All the longitudinal samples were first screened using microarray 1 as previously described, with minor modifications (34). Serum was first diluted 1:50 with serum dilution buffer (1% bovine serum albumin,1% casein, 0.5% sucrose, 0.2% polyvinylpyrrolidone, 0.5% Tween 20 in 0.01 M phosphate-buffered saline, pH 7.4), and 200 μl diluted serum was added into each microarray and incubated for 30 min on a shaker (150 rpm, 22°C). A microarray incubated with serum dilution buffer was used as a negative control. The microarray was then rinsed 3 times with washing buffer and incubated with 200 μl of horseradish peroxidase (HRP)-conjugated rabbit anti-goat IgG (Sigma-Aldrich) diluted 1:20,000 in peroxidase conjugate stabilizer/diluent (Thermo Scientific) for another 30 min on a shaker (150 rpm, 22°C), followed by the same washing steps as described above. Twenty-five microliters of chemiluminescence substrate (Thermo Scientific) were added onto the microarray, and the images were taken at a wavelength of 635 nm using the Clear 4 imaging system (Suzhou Epitope, China). The images were analyzed with Matlab. The signal of any peptide dot was defined as the signal readout of dots minus the signal readout of background.

### cELISA.

cELISA (IDVet) was performed according to the manufacturer’s instructions. Briefly, 50-μl volumes of samples diluted 1:2 were added into the 96-well plate, where positive and negative controls were in duplicate, respectively. The plate then was incubated for 45 min ± 4 min at 37°C (±3°C) and followed by washing 3 times with wash buffer. One hundred microliters of conjugate (1×) were added into the wells and incubated for another 30 min ± 3 min at 21°C (±5°C) followed by washing 3 times again. One hundred microliters of substrate were added into the wells and incubated for 15 min ± 2 min in dark at 21°C (±5°C), followed by the addition of 100 μl of stop solution. Finally, the absorbance of each well was read at 450 nm using a microplate reader.

### VNT.

The PPRV was diluted to 102.0 TCID_50_/0.1 ml with serum-free cell culture medium (MEM); the serums from the test group, positive control group, and negative group were inactivated in a water bath at 56°C for 30 min. The nonimmunized goat serum and negative serum were diluted 1:2, 1:4, and 1:8 with MEM, respectively. The immunized goat serum and positive control serum were first diluted 1:4 and then made into 2-fold serial dilutions to 1:32. Each dilution of the serum was separately added to 5 wells of a 96-well plate (0.1 ml/well). A virus regression experiment was also established. The virus working solution with 100 TCID_50_/0.1 ml was diluted with MEM to 10 TCID_50_/0.1 ml, 1 TCID_50_/0.1 ml, and 0.1 TCID_50_/0.1 ml. Each gradient virus solution was inoculated into 5 wells (0.1 ml/well). We added 0.1 ml of 100 TCID_50_/0.1 ml of virus working solution to all serum wells; 0.2 ml of MEM was added to the cell control well, 0.1 ml of MEM was added to the virus control well, and the mixture was allowed to incubate at 37°C for 1 h. Next, we added 0.1 ml of Vero cell suspension with a concentration of 200,000 to 300,000 cells/ml to all wells and incubated for 6 days at 37°C in a 5% CO_2_ incubator. The plates were checked periodically using an inverted microscope, and observation of a cytopathic effect (CPE) meant that the PPRV had not been neutralized by the serum dilution. Serum was considered positive for PPRV antibodies if the neutralizing dilution was greater than or equal to 1:10.

### Blocking test.

The blocking test (Fig. 3C) was performed as previously reported with minor modifications (35). First, the lyophilized peptides were dissolved with 30% acetonitrile solution (vol/vol, in pure water) to 1 mg/ml and further diluted to 0.5 mg/ml with serum dilution buffer; the proteins N, F, and H were diluted to 0.2 mg/ml with serum dilution buffer. The serum T3-2 (18 dpv) diluted 1:100 with dilution buffer was mixed with an equal volume of the diluted peptide or protein solution and incubated on a shaker for 30 min at room temperature (r.t.). One hundred microliters of the mixed solution were then transferred to microarray 2 and incubated for 30 min (500 rpm, 37°C), rinsed 3 times with washing buffer, and incubated with of rabbit anti-goat IgG-HRP diluted 1:20,000 in peroxidase conjugate stabilizer/diluent for another 30 min (500 rpm, 37°C), followed by the same washing step. Fifteen microliters of chemiluminescence substrate (Thermo Scientific) were added onto the microarray, and the images were taken at a wavelength of 635 nm using the Clear 4 imaging system. The images were analyzed with Matlab.

### Denaturing treatment.

One hundred microliters of 8 M urea or a combination of 8 M urea and 20 mM DTT were added to the microarray 2 to incubate for 30 min at r.t. (Fig. 3A). Then the microarray was rinsed 3 times with washing buffer and incubated with specific serum samples, followed by the steps described in the blocking test.

### Field samples tested with microarray 2 (verification/validation groups).

Large-scale field samples were tested with microarray 2. One hundred microliters of serum sample diluted 1:50 were incubated for 30 min (500 rpm, 37°C), rinsed 3 times with washing buffer, and incubated with of rabbit anti-goat IgG-HRP diluted 1:20,000 in peroxidase conjugate stabilizer/diluent for another 30 min (500 rpm, 37°C), followed by the same washing step. Seventy microliters of TMB (Thermo Scientific) were added onto the microarray and stood for 5 min to produce insoluble dark-blue precipitate, followed by washing with pure water and finally imaging by a scanner (ABT-XS01 imaging system, Suzhou Epitope, China) for chromogenic substrate. A positive control serum (PCS), responsive to all ECSPs and proteins, and a negative control (NCS) serum were included in the test, and the signal of individual ECSPs or proteins acquired by Matlab was further converted to the ratio of sample to positive control (S/P) using the following formula: S/P = (signal [sample] – signal [NCS])/(signal [PCS] – signal [NCS]). All the tests involving wild virus-infected sera were performed in biosafety level 3 laboratory in the Lanzhou Veterinary Research Institute, Chinese Academy of Agricultural Sciences.

### Statistical analysis.

The R package pheatmap was used for the cluster analysis and heat map created. The ROC curve was created with GraphPad Prism software.
